# Kidney-Targeted Redox Scavenger Therapy Prevents Cisplatin-Induced Acute Kidney Injury

**DOI:** 10.3389/fphar.2021.790913

**Published:** 2022-01-03

**Authors:** Ryan M. Williams, Janki Shah, Elizabeth Mercer, Helen S. Tian, Vanessa Thompson, Justin M. Cheung, Madeline Dorso, Jaclyn M. Kubala, Lorraine J. Gudas, Elisa de Stanchina, Edgar A. Jaimes, Daniel A. Heller

**Affiliations:** ^1^ The City College of New York Department of Biomedical Engineering, New York, NY, United States; ^2^ Memorial Sloan Kettering Cancer Center, New York, NY, United States; ^3^ Weill Cornell Medical College, New York, NY, United States; ^4^ Department of Pharmacology, Weill Cornell Medical College, New York, NY, United States

**Keywords:** nanomedicine, pharmacology, acute kidney injury, drug repurposing, cisplatin, redox scavenger

## Abstract

Cisplatin-induced acute kidney injury (CI-AKI) is a significant co-morbidity of chemotherapeutic regimens. While this condition is associated with substantially lower survival and increased economic burden, there is no pharmacological agent to effectively treat CI-AKI. The disease is hallmarked by acute tubular necrosis of the proximal tubular epithelial cells primarily due to increased oxidative stress. We investigated a drug delivery strategy to improve the pharmacokinetics of an approved therapy that does not normally demonstrate appreciable efficacy in CI-AKI, as a preventive intervention. In prior work, we developed a kidney-selective mesoscale nanoparticle (MNP) that targets the renal proximal tubular epithelium. Here, we found that the nanoparticles target the kidneys in a mouse model of CI-AKI with significant damage. We evaluated MNPs loaded with the reactive oxygen species scavenger edaravone, currently used to treat stroke and ALS. We found a marked and significant therapeutic benefit with edaravone-loaded MNPs, including improved renal function, which we demonstrated was likely due to a decrease in tubular epithelial cell damage and death imparted by the specific delivery of edaravone. The results suggest that renal-selective edaravone delivery holds potential for the prevention of acute kidney injury among patients undergoing cisplatin-based chemotherapy.

## Introduction

Acute kidney injury (AKI) is a common clinical condition associated with significant morbidity and mortality regardless of etiology or setting. AKI affects millions of individual patients and has a large socioeconomic impact, including longer hospital stay and higher costs. In the United States alone, it is estimated that the annual costs related to AKI are up to $24 billion (Silver and Chertow, 2017). The incidence of AKI is increasing at a rapid pace (Xue et al., 2006; Goldberg and Dennen, 2008), which is attributable to several factors including shifts in demographics, severity of underlying diseases, and expansion of invasive and complex medical procedures (Hoste and Schurgers, 2008; Zappitelli, 2008). AKI can result from a variety of insults including volume depletion, septicemia, hypotension, and commonly used drugs including antibiotics and chemotherapeutic agents.

Cisplatin is a widely used chemotherapy in the treatment of a variety of cancers including ovarian, head and neck, bladder, testicular, and lung, among others (Belani, 2004; Latcha et al., 2016). A significant side effect of cisplatin therapy is AKI which is seen in approximately 33% of patients (Ozkok and Edelstein, 2014; Latcha et al., 2016). Importantly, the development of AKI in these patients can result in the interruption of chemotherapy or a change to less effective chemotherapies (Ozkok and Edelstein, 2014; Latcha et al., 2016). There is therefore a critical need to develop novel strategies to prevent or treat AKI induced by cisplatin, which would also potentially have a direct impact on the oncologic outcomes of these patients whose treatment otherwise cannot be completed, or other less-effective chemotherapeutic agents must be used (Al-Mamgani et al., 2017).

There has been significant overall progress in our understanding of the epidemiology, pathophysiology and outcomes of AKI. Despite such substantial advances*,* almost no meaningful progress has been made in the treatment of AKI and there are no effective pharmacologic approaches currently approved for the treatment of AKI (Bellomo et al., 2012). Hence, clinicians rely on conservative measures and renal replacement therapy if indicated for the management of AKI (Greenberg and Cheung, 2005). Numerous strategies have been proposed to prevent, ameliorate, or treat AKI. These include inhibition of inflammatory mediators, enhancement of renal perfusion by blocking vasoconstrictor mechanisms and/or enhancing vasodilation, attenuation of leukocyte infiltration, inhibition of the coagulation cascade, inhibition of reactive oxygen species (ROS), and administration of growth factors to accelerate renal recovery (Zarjou et al., 2012; Humphreys et al., 2015). Most of these attempts have shown moderate success in attenuating AKI in animal models but have subsequently failed in clinical trials. The reasons for these failures are multifactorial but often include poor delivery of therapeutic agents to the proximal tubules, systemic toxicity at doses required to have a therapeutic effect, concomitant co-morbid conditions, suboptimal clinical trial design, and heterogeneity in cause and timing of AKI, among others (Awdishu and Joy, 2016).

We previously developed a mesoscale nanoparticle (MNP) (Williams et al., 2015; Williams et al., 2018), a drug carrier that localizes therapeutic and imaging payloads to the kidneys. The nanoparticles specifically target the renal cortex tubular epithelium, localizing in both proximal tubular and distal tubular epithelial cells. In our prior work, we investigated the pharmacokinetics and mechanism of renal localization of the particles themselves *via in vivo* and *ex vivo* imaging experiments (Williams et al., 2015; Williams et al., 2018). We found that renal targeting of MNPs depended on their size (350–400 nm) and surface chemistry (polyethylene glycol-coated surface) (Williams et al., 2015; Williams et al., 2018), whereas other sizes or surface modifications exhibited primarily liver accumulation. These studies utilized MNPs loaded with a fluorescent dye and found that the particles are likely too large for glomerular filtration and reabsorption through the luminal membrane of tubular epithelial cells. Instead, they suggested that particles reach the tubular epithelial cells *via* transcytosis across the peritubular capillaries and were taken up by tubular epithelial cells at their basolateral membrane, similar to previously-studied mechanisms (Stamatiades et al., 2016). As these particles have no specific targeting moiety for the peritubular endothelium or tubular epithelium, we hypothesized that this uptake was primarily driven by a decrease in fluid flow in the peritubular capillary combined with a large increase in absorptive pressure of peritubular capillaries (Brenner et al., 1971; Aird, 2007). Further, the particles were found to be biodegradable, releasing cargoes over days to weeks, and they exhibited minimal toxicity (Williams et al., 2018). The MNP drug carrier platform has been investigated in ischemia-reperfusion injury, using experimental drug payloads including peptides and oligodinucleotides (Han et al., 2020a; Han et al., 2020b).

No clinical methods currently exist to target the majority of a therapeutic specifically to the site of CI-AKI (Jo et al., 2007; Williams et al., 2016). Most attempted therapeutic strategies for CI-AKI have been hindered by side effects and/or poor drug accumulation at the site of injury (Walz et al., 2010; Di Lorenzo et al., 2011; Gewin et al., 2012). Reactive oxygen species (ROS) scavenger therapy, for instance, has been investigated in the treatment of CI-AKI, as oxidative stress is a predominant mechanism of injury in cisplatin-induced AKI (Ozkok and Edelstein, 2014; Golombek et al., 2018), but successful attempts required high doses to effect efficacious responses in rodents (Iguchi et al., 2004), suggesting that human trials would result in dose-limiting toxicities. If strategies existed to change drug pharmacokinetics to localize therapies to the proximal renal tubules, therapeutic efficacy would likely be significantly improved (Zarjou et al., 2012). Additionally, it is hypothesized that therapeutic intervention proximal to the time of cisplatin infusion would be most efficacious in the treatment of CI-AKI, due to the rapid onset of tubular necrosis after cisplatin dosing and the poor timing of AKI diagnosis and therapy initiation (Walz et al., 2010; Di Lorenzo et al., 2011; Gewin et al., 2012). The pathophysiology of CI-AKI therefore warrants preventive therapy, if the intervention were to exhibit minimal risk of toxicities.

Here, we investigated the potential for preventive therapy of CI-AKI *via* kidney-targeted delivery of free radical scavenger treatment. The free radical scavenger edaravone, approved in the United States for ALS and in Japan for ischemic stroke and ALS (Lapchak, 2010; Voelker, 2017), was encapsulated in the MNP drug carrier. We found that edaravone-loaded MNPs targeted the kidneys in a CI-AKI mouse model, improved the pharmacokinetics of edaravone, and imparted substantial protection against CI-AKI as measured by renal function, histology, and oxidative stress levels.

## Materials and Methods

### MNP Formulation

Mesoscale nanoparticles were formulated from a di-block PLGA-PEG copolymer similarly to described in our prior work (Williams et al., 2015; Williams et al., 2018). PLGA-PEG was synthesized *via* conjugation of carboxylic acid-terminated poly(lactic-*co*-glycolic acid) (PLGA) and heterobifunctional polyethylene glycol (NH_2_-PEG-COOH). PLGA (50:50; MW 38–54 kDa; 5 g; Aldrich, St. Louis, MO) was dissolved in 10 ml methylene chloride and mixed with N-hydroxysuccinimide (NHS; 135 mg; Aldrich) and 1-ethyl-3-(3-(dimethylamino)propyl)-carbodiimide (EDC; 230 mg; Aldrich) overnight. Activated PLGA-NHS was precipitated with cold ethyl ether and washed 3× with cold 50:50 ethyl ether:methanol before drying under vacuum. Dried PLGA-NHS (1 g) was dissolved in chloroform with heterobifunctional PEG (250 mg; Nanocs, New York, NY) with 37 μl *N,N*-diisopropylethylamine and mixed overnight. PLGA-PEG was precipitated with cold methanol and washed 3× with the same before drying under vacuum. Conjugation was confirmed *via* ^1^H NMR as previously described (Cheng et al., 2007).

MNPs were formulated *via* nanoprecipitation. PLGA-PEG (100 mg) was dissolved in acetonitrile (2 ml) with either edaravone (100 mg; Santa Cruz Biotechnology, Dallas, TX) for Eda-MNPs, or with no co-precipitate molecule for Ctrl-MNPs. This was added dropwise (100 µl/min) to purified water (4 ml) with Pluronic F-68 (100 μl; Life Technologies, Carlsbad, CA). Each batch of particles was stirred for 2 h prior to centrifugation at 7356 RCF for 15 min prior to washing with purified water and centrifuging again. The recovered MNPs were suspended in a 2% sucrose solution and lyophilized until a fluffy powder was obtained.

### MNP Characterization

MNP batches were characterized to determine their size and charge. Size and polydispersity index (PDI) were measured *via* dynamic light scattering (DLS; Malvern, Worcestershire, United Kingdom) in 1x phosphate buffered saline (PBS) at 10 mg/ml. Surface charge was measured *via* electrophoretic light scattering (ELS; Malvern) in purified water at 1 mg/ml. The effect of serum on surface charge was determined by incubating each batch at 1 mg/ml in 100% fetal bovine serum (FBS) at room temperature for 30 min, centrifuging at 7356 RCF for 15 min, and resuspending the particle pellet in purified water for ELS. For each size and charge measurements, particle batches were measured three times and the mean ± standard deviation as reported.

Cargo loading and release were also characterized for Eda-MNPs. Cargo loading was determined by dissolving particles in acetonitrile, centrifuging at 31,000 RCF for 15 min to remove polymer, and performing UV-VIS spectrophotometry (Jasco, Easton, MD) on the supernatant. Cargo release was determined by suspending particles at 10 mg/ml PBS and incubating for 72 h at 37°C or room temperature. At 2, 4, 6, 24, and 48 h, as well as the final 72 h timepoint, particles were centrifuged and the supernatant measured *via* UV-VIS spectrophotometry.

### Cisplatin-Induced AKI Mouse Model

To next investigate the therapeutic efficacy of Eda-MNPs, we recapitulated a model of cisplatin-induced acute kidney injury as previously described (Kim et al., 2012). Briefly, male 8–12-weeks C57BL/6 mice (Charles River, Troy, NY) were deprived of food and water for 18 h prior to induction. We used male mice only as female mice are more resistant to renal injury (Müller et al., 2002; Wei et al., 2005; Yang et al., 2010). Cisplatin (Sigma) was prepared for injection by dissolving at 1 mg/ml in sterile saline followed by a 30-min incubation in a 37°C water bath to ensure dissolution while protecting from light. Mice were then injected intraperitoneally with 25 mg/kg, at which time food and water were returned. Mice were sacrificed 72 h following cisplatin injection following a terminal retroorbital bleed. In addition to serum collection, kidneys were harvested and prepared for further study. Healthy control groups either underwent fasting and dehydration with no cisplatin injection or received food and water *ad libitum*.

### Therapeutic Effect of Eda-MNPs in CI-AKI

We then sought to determine the therapeutic efficacy of Eda-MNPs in mice with CI-AKI. We investigated six total mouse groups (*N* = 5–10), including negative controls, positive controls, two unencapsulated drug groups, empty MNPs, and Eda-MNPs as the investigative group: 1) Healthy control mice; 2) CI-AKI; 3) CI-AKI + 50 mg/kg Eda-MNPs (intravenous in PBS); 4) CI-AKI + 0.2 mg/kg free edaravone (intravenous in PBS; matched dose); 5) CI-AKI + 30 mg/kg free edaravone (intravenous in PBS; high dose); and 6) CI-AKI + 50 mg/kg Ctrl-MNPs (intravenous in PBS). All treatments were performed 24 h after cisplatin injection prior to sacrifice at 72 h and organ formalin fixing and staining as above. Mice were weighed at the time of food and water removal, at cisplatin administration, and every 24 h afterwards until sacrifice.

We also performed a therapeutic efficacy study in mice with CI-AKI that were not fasted and dehydrated to further confirm the potential clinical utility of this therapeutic tool. In this experiment, cisplatin was injected into mice as above, though we only investigated negative control, positive control, and investigational therapeutic groups (*N* = 3–5): 1) Healthy control mice; 2) CI-AKI; and 3) CI-AKI + 50 mg/kg Eda-MNPs. MNPs were administered *via* the tail vein 24 h after CI-AKI induction. Serum was collected for further study 72 h after cisplatin administration at the time of sacrifice.

### Mass Spectrometry Analysis of Blood and Renal Tissue

To determine the systemic and local concentrations of edaravone facilitated by MNP delivery, we performed liquid chromatography-mass spectrometry (LC-MS). Four healthy C57BL/6 male 8–10 week old mice were injected intravenously with 50 mg/kg Eda-MNPs. Separately, four additional mice were injected intravenously with 0.2 mg/kg free edaravone. Each mouse was sacrificed 3 h after dosing; plasma and kidneys were collected for analysis.

For plasma preparation, 100 µl of plasma from each mouse was protein-precipitated with 500 µl cold acetonitrile before centrifugation at 4°C for 10 min at 12,700 RPM. The supernatant was dried and resuspended in 200 µl acetonitrile for LC-MS analysis. For kidney tissue preparation, each kidney was placed into a homogenization tube with ceramic beads with 1.5 ml acetonitrile. Tubes were run for 30 s at 6 m/s on a BeadMill24 (Fisher). Tubes were cooled on ice for 5 min prior to centrifugation at 12,700 RPM for 15 min at 4°C. The supernatant was dried and combined prior to completion. Following complete drying, 200 µl of acetonitrile was added and vortex for 10 min prior to centrifugation under the same condition. The supernatant was carried on to LC-MS analysis.

Both sets of samples were run on a Shimadzu LCMS-8030 triple quadrupole LC-MS (Kyoto, Japan). Protocol validation determined edaravone MRM transitions: 175 → 65.05 (quant), 175 → 44.05, and 175 → 77.05. Solvent conditions were 0.1% formic acid in HPLC-grade water for the aqueous phase and 0.1% formic acid in methanol for the organic phase. Edaravone-spiked plasma calibrations were performed, with linear values ranging from 10 ng/ml to 10 μg/ml. Limit of quantification values were 10 ng/ml in plasma and approximately 7.6 pg/mg in kidney tissues, based on an average of kidney weights (approximately 261 mg combined).

### Renal Function Analysis

At the time of animal sacrifice, blood was collected in serum preparation tubes (Becton Dickinson, Franklin Lake, NJ) and processed per manufacturer’s recommendation. Serum was analyzed for urea nitrogen and creatinine levels *via* colorimetric methods in the MSKCC Laboratory for Comparative Pathology. For CI-AKI therapeutic statistical analysis, a one-way ANOVA with Sidak’s posttest to compare to the cisplatin group was performed.

### Renal Histological Analysis

Kidneys were formalin-fixed and prepared for histological analysis. Tissues were dehydrated and embedded in paraffin prior to obtaining 5 µm sections, placing on a glass slide, and deparaffinizing. Sections were heat-retrieved at pH 6 for 20 min and peroxide-blocked for 10 min. Slides were incubated with each antibody or reagent below (1:400; Abcam, Cambridge, United Kingdom) for 50 min and then incubated with a biotinylated secondary antibody for 8 min (Vector Laboratories, Burlingame, CA). Slides were incubated with an anti-rabbit-conjugated horseradish peroxidase (HRP; Leica, Wetzlar, Germany) for 8 min and colorized with 3,3’-diaminobenzidine (DAB; Ventana Medical Systems, Tuscon, AZ). Haematoxylin (Ventana Medical Systems) was used to counterstain slides prior to Permount (Fisher Scientific, Waltham, MA) coverslipping. Digital slide images were obtained with a Leica AT2 slide scanner.

A portion of renal tissue from each group above was formalin-fixed and paraffin embedded as described above for histological analysis. Five parallel sections were obtained for each animal and subjected to the following treatments. 1) Hematoxylin and eosin (H&E) (MSKCC Molecular Cytology Core Facility or HistoWiz Inc.); 2) Polyethylene-glycol (PEG) immunohistochemistry (IHC) to confirm particle localization (HistoWiz Inc., Brooklyn, NY) 3) NGAL IHC (HistoWiz); 4) Nitrotyrosine IHC (MSKCC Core); 5) p53 IHC (MSKCC core); 6) Terminal deoxynucleotidyl transferase-dUTP nick end labeling (TUNEL) IHC (MSKCC Core).

We performed semi-quantitative image analysis of 3-nitrotyrosine staining using ImageJ Fiji similarly to as previously described for at least five individuals per group (Schindelin et al., 2012; Crowe and Yue, 2019). Briefly, digital slide images were captured at ×5 magnification to include both renal cortex and medulla then opened in Fiji. Each file underwent color deconvolution using the “H DAB” vector option. The DAB vector for each image was then set to the same minimum and maximum threshold values to remove noise and ensure consistency. Measurements of the mean grey value were obtained for each thresholded vector, which was then plotted for each group. Measurements were subjected to a one-way ANOVA with Sidak’s posttest to compare each group to the positive cisplatin control group.

## Results and Discussion

### MNP Formulation and Characterization

To specifically deliver the free radical scavenger edaravone to the site of CI-AKI, we formulated kidney-targeted mesoscale nanoparticles to encapsulate the drug *via* nanoprecipitation as previously described (Williams et al., 2015; Williams et al., 2018). We formulated edaravone with the block-copolymer poly(lactic-co- glycolic) acid (PLGA) conjugated to acid-terminated polyethylene glycol (PEG), or PLGA-PEG, obtaining therapeutic MNPs (Eda-MNPs). We also produced control MNPs with no encapsulated molecules *via* essentially the same procedure, apart from a lack of co-precipitate small molecule (Ctrl-MNPs).

**FIGURE 1:**
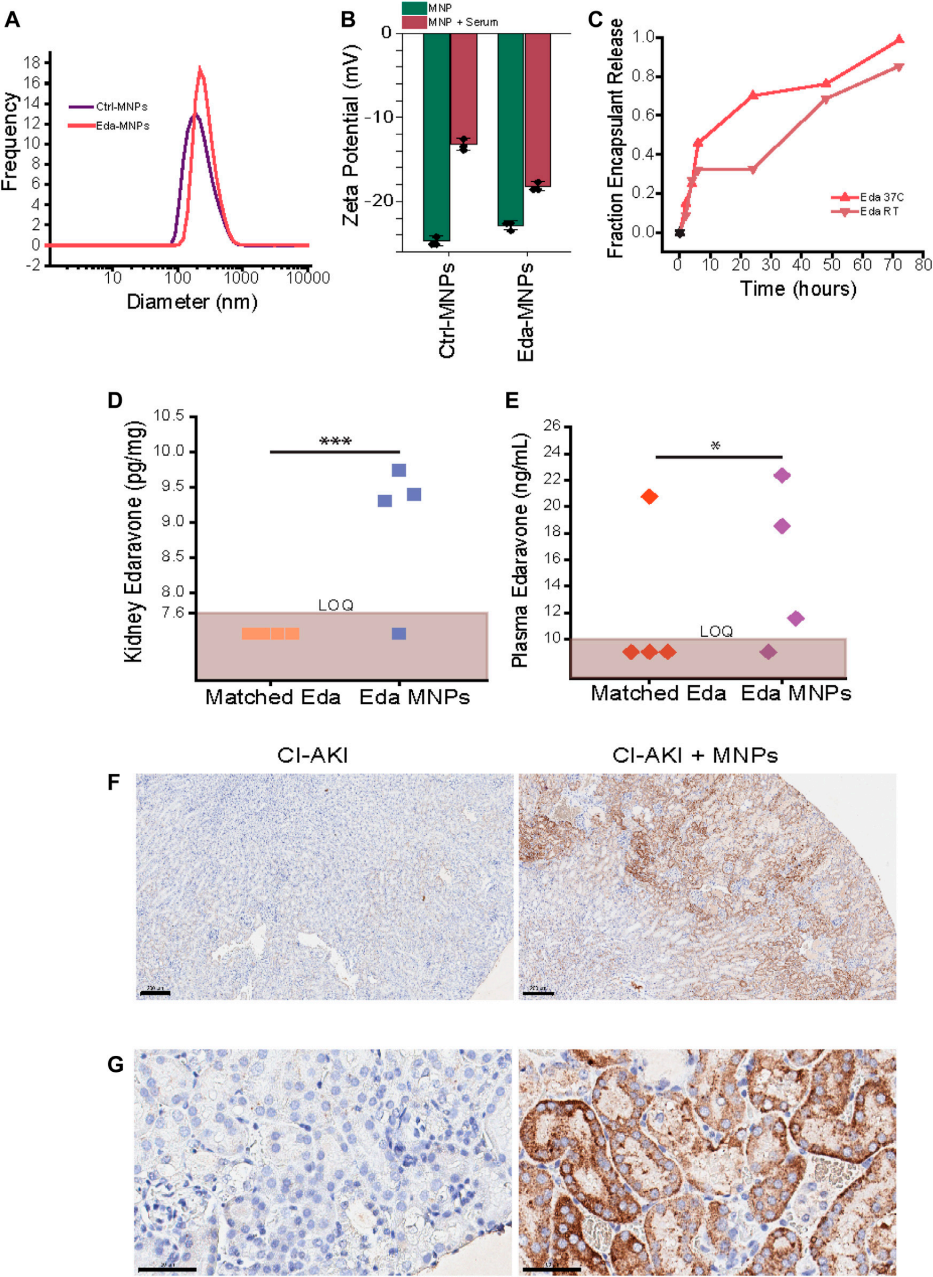
Encapsulation and kidney-selective delivery of edaravone by polymeric mesoscale nanoparticles. **(A)** Hydrodynamic diameter distribution of Ctrl-MNPs and Eda-MNPs used in all studies. **(B)** ζ-potential of MNPs used in all studies in water versus after incubation with fetal bovine serum. **(C)** Percent release edaravone at room temperature (RT) or 37°C over time. **(D)** LC-MS quantification of edaravone levels as a function of renal mass. LOQ represents approximately 7.6 pg/mg. Each data point represents a single mouse, and data points in the brown zone signify they were below LOQ. A chi-squared test was performed to determine the significance between the number of values above and below LOQ threshold; *p* = *** = 5.32E^−4^. **(E)** LC-MS quantification of edaravone levels as a function of total recovered plasma volume. LOQ represents approximately 10 ng/ml. Each data point represents a single mouse, and data points in the brown zone signify they were below LOQ. A chi-squared test was performed to determine the significance between the number of values above and below LOQ threshold; *p* = * = 0.021. **(F)** Representative immunohistochemistry (at ×5 magnification) with anti-PEG antibody staining for MNP accumulation in renal tissue observing part of cortex and medulla. DAB detection (brown) and counterstained with haematoxylin (blue). Scale bar = 200 µm. **(G)** ×40-magnification image of primarily cortex from panel **(F)**. Scale bar = 50 µm.

To characterize MNPs, we first performed hydrodynamic diameter and ζ-potential assays (Table 1). Eda-MNPs exhibited an average diameter (z average) of 374.0 ± 12.2 nm with a polydispersity index (PDI) of 0.337. 85% of these particles were within the 164–531 nm size range (Table 1; Figure 1A). Eda-MNPs exhibited a ζ-potential of −22.8 ± 0.55 mV in distilled water and −18.2 ± 0.51 mV after incubation with 100% serum (Figure 1B). Ctrl-MNPs exhibited an average size of 332.0 ± 7.56 nm with a PDI of 0.302. 74% of these particles fell within the 164–531 nm size range (Table 1; Figure 1A). Ctrl-MNPs exhibited a ζ-potential in distilled water of −24.7 ± 0.61 mV and −13.2 ± 0.7 mV in 100% serum (Figure 1B). In two prior studies with MNPs, we found that particles of this composition with hydrodynamic diameters of 347.6 and 386.7 nm, PDIs of approximately 0.3, and surface charges in water of approximately −20 mV each had significant renal accumulation (Williams et al., 2015; Williams et al., 2018). We also found that the negative surface potential was partially abrogated after serum incubation (Williams et al., 2015; Williams et al., 2018). Therefore, we would expect that given the similar size and charge characteristics, as well as their interaction with serum, each of the formulations would exhibit similar renal targeting behavior.

**TABLE 1:** Physical characteristics of MNPs used in all studies.

Formulation	Hydrodynamic diameter (nm)	PDI	% within 164–531 nm	ζ-potential in distilled water (mV)	ζ-potential in 100% serum (mV)
Eda-MNPs	374.0 (12.2)	0.337 (0.032)	85	−22.8 (0.55)	−18.2 (0.51)
Ctrl-MNPs	332.0 (7.56)	0.302 (0.040)	74	−24.7 (0.61)	−13.2 (0.70)

We performed *in vitro* cargo release assays to evaluate the period over which MNPs release the encapsulated drug (Figure 1C). First, we found that Eda-MNPs contained 3.9 µg edaravone per 1 mg of particle. Studies were performed in complete serum either at room temperature or 37°C, with measurements taken frequently up to 6 h and daily to 72 h. Eda-MNPs exhibited similar release characteristics at both room temperature and 37°C (Figure 1C). However, only about 30% of edaravone was released at room temperature up to 6 h and an additional 30% was released between 24 and 48 h. At elevated temperatures, the release rate was increased, though retaining the same pattern.

### Edaravone Pharmacokinetics Substantially Modulated by MNPs

We first investigated the pharmacokinetics of MNPs loaded with the redox scavenger molecule edaravone (Eda-MNPs). We performed mass spectrometry studies on renal tissue and plasma in healthy mice treated with Eda-MNPs or dose-matched free edaravone, sacrificed 3 h after injection. We found that measurable levels of edaravone (7.6 ng/ml or above) were only present in the kidneys when encapsulated within MNPs (Figure 1D). Plasma levels of edaravone indicated the same trend (Figure 1E). These studies suggest that MNPs substantively modified the pharmacokinetics of edaravone, allowing substantial localization in the kidneys while also increasing the concentration available in circulation. Next, we performed immunohistochemistry (IHC) with an anti-PEG antibody to stain for MNPs in the kidneys (Williams et al., 2015) (Figures 1F,G). In agreement with previous studies, we found that Eda-MNPs accumulate in primarily renal proximal tubular epithelial cells (Williams et al., 2015; Williams et al., 2018).

### Eda-MNPs Are Efficacious Against CI-AKI

To evaluate the potential clinical application of MNPs, we next sought to recapitulate a mouse model of CI-AKI. To do so, first we induced CI-AKI in 8–10 weeks male C57BL/6 mice *via* intraperitoneal injection of 25 mg/kg cisplatin following 18 h of fasting/water deprivation.

To evaluate the therapeutic efficacy of Eda-MNPs against CI-AKI, we injected the particles 24 h following cisplatin injection and injury initiation (Table 2). We performed additional control experiments in which we injected Ctrl-MNPs (empty) and two concentrations of free edaravone: 0.2 mg/kg to match that which was administered in MNPs and 30 mg/kg to match prior work with this drug in rat models of kidney injury (Iguchi et al., 2004). These were compared against cisplatin-only and healthy control groups. Mice tolerated Eda-MNP, Ctrl-MNP, and the dose-matched edaravone IV injections well, however after a single high dose of free edaravone, mice exhibited acute belabored breathing and sluggishness from which they eventually recovered. At 48 h after nanoparticle injection, and 72 h after cisplatin administration, we sacrificed the animals and obtained serum and kidney tissue for analysis.

**TABLE 2:** Individual mouse data from therapeutic efficacy studies (note: a negative weight loss percentage represents a weight gain).

Treatment	Age at euthanasia (Days)	Pre-dehydration weight (g)	Pre-cisplatin weight (g)	Day 1 weight (g)	Day 2 weight (g)	Day 3 weight (g)	Pre-cisplatin weight loss (%)	Day 1 weight loss (%)	Day 2 weight loss (%)	Day 3 weight loss (%)	Serum creatinine (mg/dl)	Blood urea nitrogen (mg/dl)
Healthy	94										0.16	32
Healthy	94										NA	21
Healthy	94										0.12	23
Healthy	94										0.16	22
Healthy	94										0.15	21
Healthy	103	25	24	30	28	28	4.00	−20.00	−12.00	−12.00	0.18	26
Healthy	103	27	24	27	26	27	11.11	0.00	3.70	0.00	0.15	29
Healthy	103	28	24	28	28	28	14.29	0.00	0.00	0.00	0.18	28
Healthy	103	29	26	30	31	30	10.34	−3.45	−6.90	−3.45	0.19	29
Healthy	103	27	25	26	26	26	7.41	3.70	3.70	3.70	0.20	28
Cisplatin	72	27	23	23	21	20	14.81	14.81	22.22	25.93	1.52	241
Cisplatin	72	28	24	24	23	21	14.29	14.29	17.86	25.00	0.22	111
Cisplatin	72	27	23	22	21	20	14.81	18.52	22.22	25.93	0.95	249
Cisplatin	72	26	21	20	19	18	19.23	23.08	26.92	30.77	0.35	232
Cisplatin	72	28	26	26	24	23	7.14	7.14	14.29	17.86	1.1	243
Cisplatin	86	30	26	26	24	23	13.33	13.33	20.00	23.33	0.52	198
Cisplatin	86	27	23	23	20	20	14.81	14.81	25.93	25.93	0.35	91
Cisplatin	79	28	25	24	23	22	10.71	14.29	17.86	21.43	0.45	115
Cisplatin	79	27	26	26	24	21	3.70	3.70	11.11	22.22	0.08	85
Cis + Eda MNPs	72	27	23	23	23	20	14.81	14.81	14.81	25.93	0.09	30
Cis + Eda MNPs	72	29	25	26	25	23	13.79	10.34	13.79	20.69	0.08	44
Cis + Eda MNPs	72	30	25	24	24	22	16.67	20.00	20.00	26.67	0.08	51
Cis + Eda MNPs	72	27	23	24	23	22	14.81	11.11	14.81	18.52	0.13	38
Cis + Eda MNPs	72	29	26	27	25	22	10.34	6.90	13.79	24.14	0.07	32
Cis + Eda MNPs	79	27	24	22	23	22	11.11	18.52	14.81	18.52	0.34	126
Cis + Eda MNPs	79	28	25	21	23	21	10.71	25.00	17.86	25.00	0.03	55
Cis + Eda MNPs	79	27	25	24	23	21	7.41	11.11	14.81	22.22	0.13	92
Cis + Eda MNPs	79	27	24	23	23	21	11.11	14.81	14.81	22.22	0.07	110
Cis + Eda MNPs	79	27	24	24	22	20	11.11	11.11	18.52	25.93	0.04	50
Cis + High Dose Eda	72	30	26	26	25	24	13.33	13.33	16.67	20.00	0.13	59
Cis + High Dose Eda	72	27	23	23	23	21	14.81	14.81	14.81	22.22	0.09	45
Cis + High Dose Eda	72	25	23	23	25	22	8.00	8.00	0.00	12.00	0.18	36
Cis + High Dose Eda	72	28	24	25	23	21	14.29	10.71	17.86	25.00	0.09	61
Cis + High Dose Eda	72	29	26	24	25	23	10.34	17.24	13.79	20.69	0.13	59
Cis + High Dose Eda	86	31	26	26	22	22	16.13	16.13	29.03	29.03	1.87	286
Cis + High Dose Eda	86	29	25	25	23	22	13.79	13.79	20.69	24.14	0.39	94
Cis + High Dose Eda	86	27	23	22	22	20	14.81	18.52	18.52	25.93	0.56	126
Cis + Matched Dose Eda	85	29	26	25	DEAD	DEAD	10.34	13.79	DEAD	DEAD	NA	NA
Cis + Matched Dose Eda	86	25	21	22	20	20	16.00	12.00	20.00	20.00	0.44	167
Cis + Matched Dose Eda	86	30	26	25	23	24	13.33	16.67	23.33	20.00	1	250
Cis + Matched Dose Eda	86	31	26	30	30	30	16.13	3.23	3.23	3.23	0.25	33
Cis + Matched Dose Eda	86	30	26	26	23	23	13.33	13.33	23.33	23.33	3.41	328
Cis + Matched Dose Eda	79	28	25	23	24	21	10.71	17.86	14.29	25.00	0.26	121
Cis + Matched Dose Eda	79	28	25	23	22	21	10.71	17.86	21.43	25.00	0.1	61
Cis + Matched Dose Eda	79	27	23	23	21	20	14.81	14.81	22.22	25.93	0.08	68
Cis + Matched Dose Eda	79	29	25	22	21	20	13.79	24.14	27.59	31.03	0.23	99
Cis + Matched Dose Eda	79	30	26	24	23	21	13.33	20.00	23.33	30.00	0.11	90
Cis + Ctrl-MNPs	82	26	23	22	22	20	11.54	15.38	15.38	23.08	0.92	182
Cis + Ctrl-MNPs	82	25	21	22	20	19	16.00	12.00	20.00	24.00	1.9	231
Cis + Ctrl-MNPs	82	27	25	25	23	21	7.41	7.41	14.81	22.22	0.25	122
Cis + Ctrl-MNPs	82	26	22	23	22	21	15.38	11.54	15.38	19.23	0.45	130
Cis + Ctrl-MNPs	82	25	21	22	21	19	16.00	12.00	16.00	24.00	1.66	204

**FIGURE 2:**
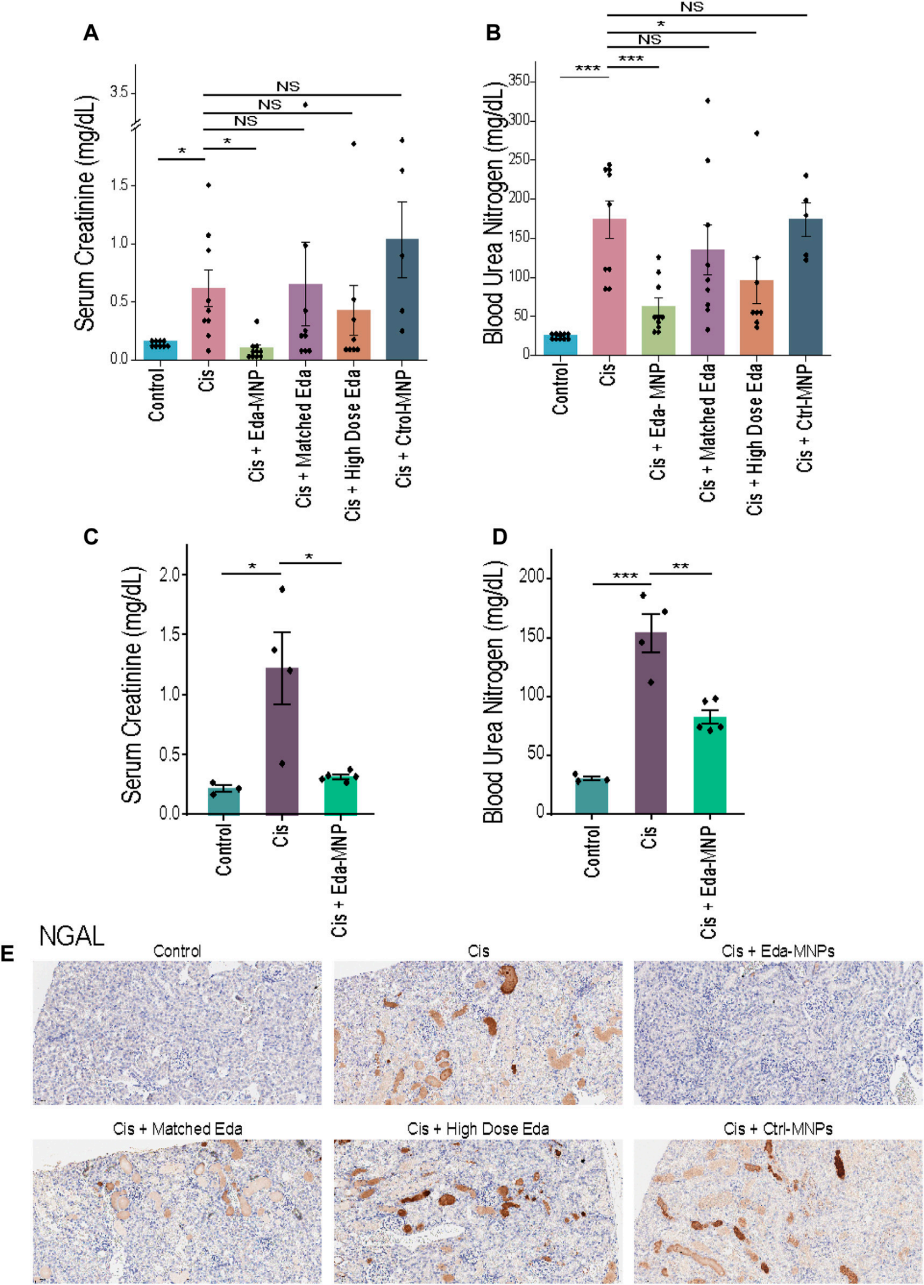
Evaluation of renal function following treatment with Eda-MNPs and controls. **(A)** Serum creatinine of mouse groups induced to have CI-AKI (Cis) and treated with Eda-MNPs or various controls. Bars represent the mean ± SEM. One-way ANOVA with Sidak’s posttest to compare to cisplatin control group. NS = *p* > 0.05; * = *p* < 0.05 **(B)** Blood urea nitrogen (BUN) of the same mouse groups as in panel **(A)**. NS = *p* > 0.05; * = *p* < 0.05; *** = *p* < 0.001. **(C)** In non-fasted mice, serum creatinine of mouse groups with no AKI (*N* = 3), induced to have CI-AKI (Cis) (*N* = 4), or treated with Eda-MNPs (*N* = 5). Bars represent the mean ± SEM. One-way ANOVA with Sidak’s posttest to compare to cisplatin control group. Control vs Cis: 0.21 ± 0.029 vs 1.22 ± 0.303 mg/dl, *p* = * = 0.013; Cis vs Cis + Eda-MNP: 1.22 ± 0.303 vs 0.31 ± 0.018 mg/dl, *p* = * = 0.012 **(D)** In non-fasted mice, blood urea nitrogen (BUN) of the same mouse groups as in panel **(C)**. One-way ANOVA with Sidak’s posttest to compare to cisplatin control group. Control vs Cis: 30.33 ± 1.86 vs 154 ± 16.27 mg/dl, *p* = *** = 0.000027; Cis vs Cis + Eda-MNP: 154 ± 16.27 vs 82.6 ± 5.91 mg/dl, *p* = ** = 0.0074. **(E)** IHC of NGAL expression in representative kidney sections from animals with each noted treatment, DAB detection (brown) and counterstained with haematoxylin (blue) (×10 magnification).

We found that mice with CI-AKI treated with Eda-MNPs had kidney function and injury biomarkers similar to those in healthy control mice. We found that these mice had significantly lower serum creatinine (SCr) levels compared to untreated CI-AKI mice with levels at the baseline for healthy mice (Figure 2A; Table 2). We also found that the two free edaravone doses (dose-matched and high dose) did not have a significant effect on SCr levels, nor did the Ctrl-MNPs (empty). We also evaluated renal health through blood urea nitrogen (BUN) levels, finding the same evidence of therapeutic efficacy from Eda-MNPs (Figure 2B; Table 2), with only the high dose of free edaravone showing a moderate statistically-significant benefit. It is clear from these serum-based markers of renal function that Eda-MNPs exhibit substantial benefit to renal function following cisplatin insult.

To further evaluate Eda-MNP efficacy, we performed a similar experiment in a different CI-AKI mouse model, in which the mice were not fasted or water deprived. Using the same Eda-MNP formulation, we again found a four-fold decrease in serum creatinine and two-fold decrease in BUN compared to mice receiving cisplatin only (Figures 2C,D).

Finally, we evaluated expression of neutrophil gelatinase-associated lipocalin (NGAL), a specific biomarker for acute kidney injury (Mishra et al., 2003; Supavekin et al., 2003). To do so, we performed IHC staining with an NGAL-specific antibody to determine expression in each group (Figure 2E). Using this metric, we found substantially less evidence of AKI in mice treated with Eda-MNP compared to mice treated with cisplatin alone or nanoparticle-alone or free drug controls. These results concur with the creatinine and BUN findings, demonstrating almost baseline levels NGAL expression in treated animals.

### Histological Analyses of Tubular Injury and Cell Death

**FIGURE 3:**
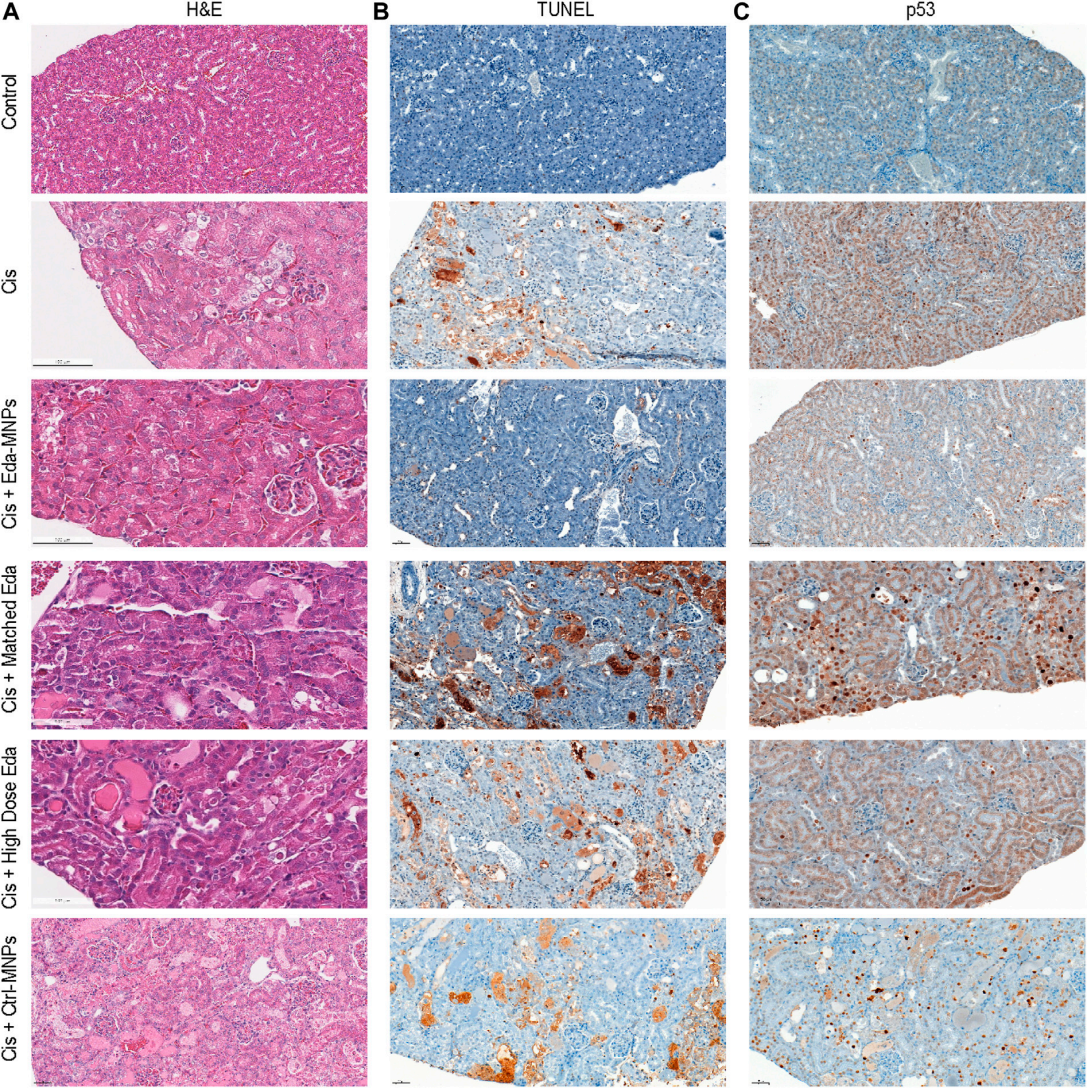
Evaluation of cell injury and death following treatment. **(A)** Renal tissues were stained with haematoxylin (blue) and eosin (pink) (H&E). **(B)** TUNEL detection in renal tissue *via* DAB (brown) and counterstained with haematoxylin (blue). **(C)** IHC with DAB detection (brown) of an anti-p53 antibody and counterstained with haematoxylin (blue) in renal tissue (×10 magnification).

To further investigate the mechanism of Eda-MNPs therapeutic benefit on CI-AKI animals, we evaluated tubular injury and cell death *via* histology. First, we analyzed hematoxylin and eosin (H&E) stains of each tissue, finding substantially reduced acute tubular necrosis, improved renal tubular architecture, and overall reduced evidence of AKI in mice treated with Eda-MNPs (Figure 3A). Next, we performed TUNEL staining to identify DNA fragmentation and apoptosis hallmarks of AKI (Havasi and Borkan, 2011) (Figure 3B), and IHC for p53 to identify loci of DNA damage and repair, overexpressed in AKI (Supavekin et al., 2003; Zhang et al., 2014) (Figure 3C). These findings clearly demonstrate an absence of apoptosis and DNA damage in mice treated with Eda-MNPs. This evidence suggests that an absence of tubular cell death was the product of tubular-specific delivery of edaravone afforded by Eda-MNPs and contributed to improved renal function.

### Pharmacodynamic Analysis of Kidney-Targeted Free Radical Scavenger Therapy

**FIGURE 4:**
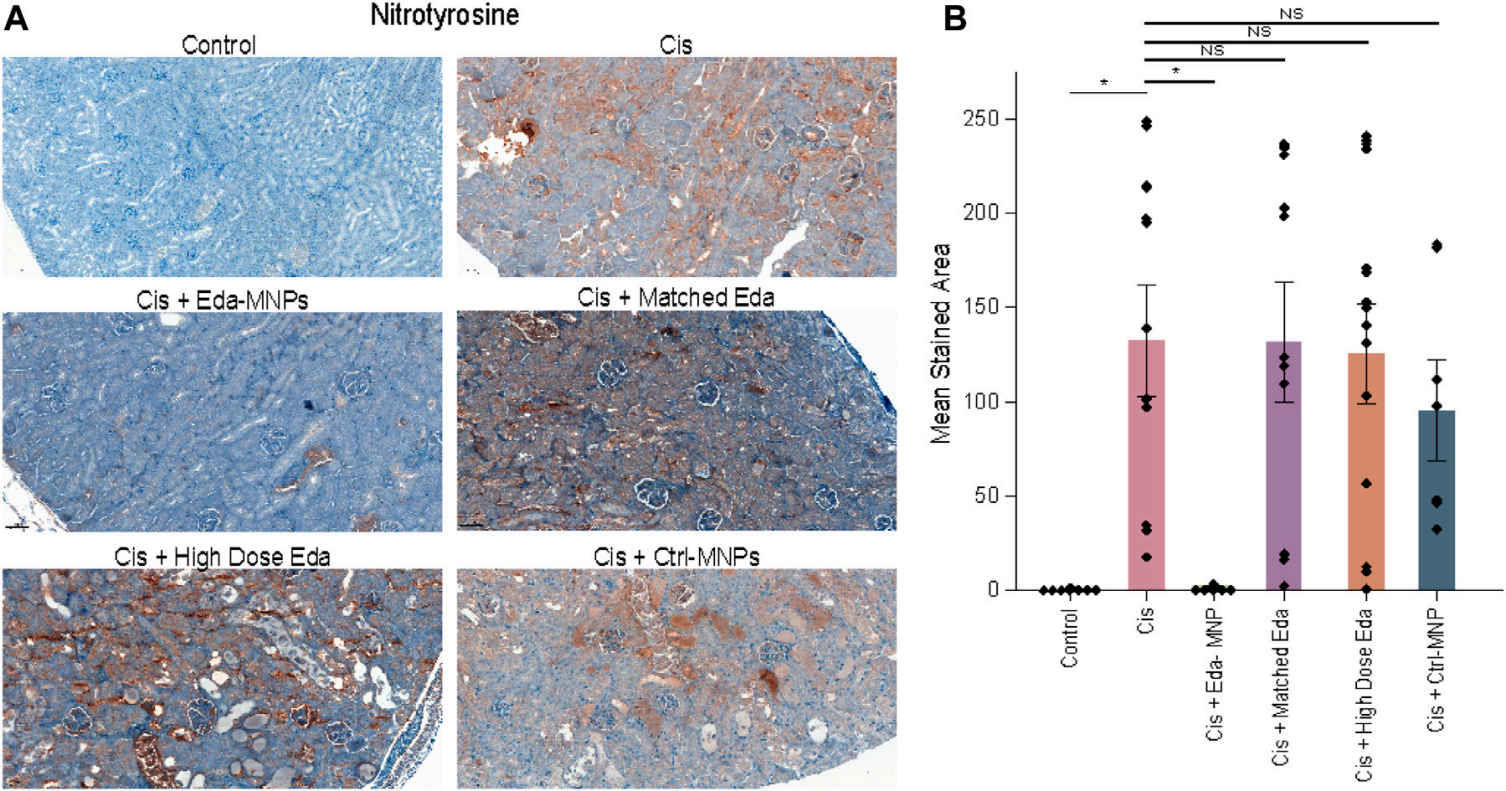
Nitrotyrosine staining to evaluate pharmacodynamic outcomes of oxidative stress following Eda-MNP therapy. **(A)** IHC with DAB detection (brown) for 3-nitrotyrosine IHC and counterstained with haematoxylin (blue) (×10 magnification). **(B)** Semi-quantitative analysis of images from each treatment group. Bars represent mean ± SEM. NS = *p* > 0.05; * = *p* < 0.05. Control vs Cis: 0.502 ± 0.203 vs 132.51 ± 29.61, *p* = * = 0.013. Cis vs Cis + Eda MNP: 132.51 ± 29.61 vs 2.094 ± 0.594, *p* = * = 0.036.

Finally, we sought to evaluate the hypothesis that a reduction of cisplatin-induced oxidative stress due to tubular-specific delivery of edaravone was the mechanism of therapeutic function. To do so, we evaluated the presence of 3-nitrotyrosine, which is the end-product of cisplatin-induced peroxynitrite formation in AKI and has previously been used to evaluate therapeutic outcomes involving this free radical pathway (Fiaccadori et al., 2004; Pan et al., 2014; Morigi et al., 2015). Nitrotyrosine has also previously been used as a pharmacodynamic endpoint for edaravone treatment in ALS clinical trials, as well as other laboratory investigations (Zhang et al., 2005; Yoshino and Kimura, 2006; Xi et al., 2007; Bandookwala et al., 2019). In addition, nitrotyrosine is widely recognized to be the direct result of cisplatin-induced cell and tissue damage, including ototoxicity and neurotoxicity in addition to nephrotoxicity (Giridharan et al., 2012; Rathinam et al., 2015; Jamesdaniel et al., 2016). Nitrotyrosine is also used as a primary endpoint with elevated levels in clinical and laboratory studies of both AKI (Thuraisingham et al., 2000; Qian et al., 2013; Sureshbabu et al., 2018) and other renal diseases (Thuraisingham et al., 2000; Fiaccadori et al., 2004). We therefore performed IHC for nitrotyrosine to evaluate the pharmacodynamics of free radical scavenger therapy. Representative IHC images showed that mice with CI-AKI had high levels of nitrotyrosine staining, whereas normal healthy mice had little to no staining (Figure 4A). Similar to prior results on renal function and cell death, we found that CI-AKI mice that received Eda-MNPs exhibited almost baseline levels of nitrotyrosine production. We confirmed these representative results by performing semi-quantitative analysis of nitrotyrosine staining (Figure 4B). This data suggests that the MNP-enapsulated free radical scavenger conferred the intended effect at the site of injury. In free edaravone controls, little ROS scavenger likely reached the site of injury.

## Conclusion

In these studies, we determined that edaravone-loaded MNPs exhibit striking therapeutic efficacy in a mouse model of CI-AKI. We determined that this response was due to a reduction of proximal tubular cell death and damage as a result of edaravone-induced free radical scavenging, only imparted by kidney-selective delivery by MNPs. We propose that, by administering edaravone *via* MNPs and not systemically, we would avoid interference with cancer treatment, as reactive oxygen species scavengers would do in cisplatin-treated patients (Sugihara and Gemba, 1986). We cannot discount, however, the possibility that some nanoparticle accumulation in the tumors may occur *via* the enhanced permeability and retention effect (EPR) and cause a reduction in cisplatin anti-tumor efficacy (Matsumura and Maeda, 1986; Sugihara and Gemba, 1986; Bertrand et al., 2014; Wang, 2015). Thus, additional studies will be necessary to evaluate to what extent this may occur, if any, though EPR-dependent nanoparticle uptake typically relies on smaller-sized particles than those studied here (Golombek et al., 2018). Though we hope that by reducing renal complications, we also would allow the patient to stay on the front-line cisplatin therapy for longer periods of time at higher doses, extending the chemotherapy’s therapeutic window and effectiveness (Go and Adjei, 1999). This advancement has clear utility in clinical cancer management and nephrology.

Edaravone (marketed under the name Radicut) was originally developed in the 1980s as a treatment for stroke (Abe et al., 1988) and has been on the market in Japan since 2001 for this indication (Group, 2003). It has been approved in Japan for ALS treatment since 2015 and in the United States since 2017 (Lapchak, 2010; Voelker, 2017). There have been several additional studies to evaluate the therapeutic efficacy of edaravone in other diseases, including traumatic brain injury and optic nerve injury, among others (Akiyama et al., 2017; Bailly, 2019; Shakkour et al., 2021). One such investigation found a moderate reduction (approximately 50% for creatinine and BUN) of AKI in a rat model of cisplatin-induced AKI (Iguchi et al., 2004). This data indeed supports our current findings from the “High-Dose Eda” free drug group, which found approximately a 33% reduction in creatinine and BUN (though only BUN was statistically significant). Minor variability may have arisen due to the species used (rat vs mouse in our studies) and the dose of cisplatin (5 vs 25 mg/kg in our studies), among other differences. However, we believe that the marked increase in efficacy afforded by MNP-based kidney-targeted delivery of edaravone marks a substantial step toward clinical utility of edaravone use in CI-AKI.

There have been few prior efforts to develop kidney-targeted drug carriers for renal disease, with even less focus on CI-AKI (Alidori et al., 2016; Jiang et al., 2018). These studies further validate the need for further validation of nanomedicine approaches in the treatment of kidney disease (Williams et al., 2016). We expect similar pre-clinical pharmacokinetic studies to further the possibility of MNP clinical translation.

We found that edaravone-loaded MNPs exhibited substantial therapeutic potential in a murine model of CI-AKI. These characteristics substantially increase the likelihood of translation to the clinic for the treatment or prevention of CI-AKI in patients with cancer. While these studies focused on acute kidney injury, we anticipate future work will evaluate this potential therapeutic specifically in animal models of chronic kidney disease, including cisplatin-induced and other etiologies (Eddy et al., 2012; Landau et al., 2019). However, the striking efficacy in preventing AKI found here portends a likely prevention of cisplatin-mediated CKD development and progression (Belayev and Palevsky, 2014; Fiorentino et al., 2018). We expect this approach to ultimately improve the quality of those patients’ lives due to increased renal function after cisplatin therapy as well as a reduction in economic toll. We also expect this to allow those patients to stay on cisplatin as a front-line therapy, allowing increased survival from the underlying cancer.

## Data Availability

The original contributions presented in the study are included in the article, further inquiries can be directed to the corresponding authors.
